# The Data Linkage Hub: a Community of Practice

**DOI:** 10.23889/ijpds.v9i5.2643

**Published:** 2024-09-18

**Authors:** Esther Lewis, Rachel Huck, Sarah Cummins

**Affiliations:** 1 Office for National Statistics

## Objective and Approach

This paper discusses the Data Linkage Hub (DLH) which is a centre for data linkage excellence and aims to produce and support the delivery of high quality, linked data assets for researchers in the UK. Here, we will give an overview of the DLH’s activity as a Community of Practice for Data Linkage.

## Approach

We will discuss the different project types conducted in the DLH; the methods used ranging from bespoke linkage development to the adoption of generalised methodologies; and the customers of the DLH.

We will also discuss the tools the DLH have developed, and make available to others, to standardise, streamline and improve quality assessment of linkage. This will include clerical matching tools known as CROW and the CMS, BAT, a bias analysis tool, and a package of PySpark code specifically developed for data linkage.

In addition, we will explore the data linkage training materials we offer for different audiences and our involvement in a Data Linkage Champions Network, both increasing the awareness of data linkage and promoting best practise.

## Conclusion and Implications

The impact of the DLH’s work is far reaching, underpinning statistical outputs, research and public policy. This success is down to the creation of a community of practice bringing together experts to regularly update and discuss linkage.

